# Endoscopic intermuscular dissection, knife-assisted full-thickness resection and defect closure for early rectal cancer

**DOI:** 10.1055/a-2784-8332

**Published:** 2026-02-05

**Authors:** Petros Zormpas, Eleni Nakou, Dimitris Dimitriadis, Marianna Spinou, Margarita-Eleni Manola, Antonis Pikoulas, George Tribonias

**Affiliations:** 1 168201 Gastroenterology Department, General Hospital Korgialenio-Benakio Red Cross, Athens, Greece

Endoscopic submucosal dissection (ESD) is often limited in treating lesions with suspected deep submucosal invasion (D-SMI 1 ). To ensure a clear vertical margin, advanced techniques such as endoscopic intermuscular dissection (EID) and knife-assisted full-thickness resection (kFTR) are viable alternatives while simultaneously enabling organ preservation 2 3 . Multidisciplinary team (MDT) consultation is imperative both before and after resection, weighing the patient preference for organ preservation and regional guidelines to determine the optimal treatment path.

We present the case of a 62-year-old man referred for a 27mm Isp polyp in the lower rectum, 2 cm from the dentate line. Endoscopic resection was preferred over transanal minimally invasive surgery as the initial treatment due to the lesion’s location and the lower risk of local recurrence 4 . Due to suspected D-SMI, the initial plan was EID. However, intraoperative expansion of the intermuscular plane proved unfeasible despite repeated injections due to severe muscle retraction. Strategies for managing the muscle retracting sign, including gravitational manipulation 5 , were attempted but proved ineffective. Consequently, the decision was made to convert to a partial full-thickness resection. kFTR was performed uneventfully using a waterjet-assisted knife (Erbe Elektromedizin, Tübingen, Germany) and a hook-type knife (Olympus, Tokyo, Japan), resulting in a full-thickness defect of the bowel wall. Endoscopic suturing of the muscle defect was performed aiming to minimize post-operative morbidity, expedite recovery, and restore the bowel wall structural integrity ( Video 1 ). Histopathology confirmed en bloc and R0 resection of a moderately differentiated pT1b adenocarcinoma with 2083 μm of submucosal invasion and no other high-risk features. Following MDT discussion and consideration of the patient’s preference to avoid radical surgery and possibly a permanent stoma, adjuvant chemoradiotherapy and active surveillance were selected.

Endoscopic intermuscular dissection completed using knife-assisted full-thickness resection and defect suturing for a rectal lesion with suspected deep submucosal invasion.Video 1

Endoscopy_UCTN_Code_TTT_1AQ_2AD_3AF
